# Identification of the Homeobox Protein Prx1 (MHox, Prrx-1) as a Regulator of Osterix Expression and Mediator of Tumor Necrosis Factor α Action in Osteoblast Differentiation

**DOI:** 10.1002/jbmr.203

**Published:** 2010-08-03

**Authors:** Xianghuai Lu, George R Beck, Linda C Gilbert, Corinne E Camalier, Nicholas W Bateman, Brian L Hood, Thomas P Conrads, Michael J Kern, Shaojin You, Hong Chen, Mark S Nanes

**Affiliations:** 1 VA Medical Center and Division of Endocrinology, Metabolism, and Lipids, Department of Medicine, Emory University School of MedicineAtlanta, GA, USA; 2 Department of Pharmacology and Chemical Biology, University of PittsburghPittsburgh, PA, USA; 3 Department of Cell Biology and Anatomy, Medical University of South CarolinaCharleston, SC, USA

**Keywords:** OSTEOBLASTS, BONE, TNF, PRX1, MHOX

## Abstract

Tumor necrosis factor α (TNF-α) promotes bone loss and inhibits bone formation. Osterix (Osx, SP7) is a transcription factor required for osteoblast (OB) differentiation because deletion results in a cartilaginous skeleton. We previously described a TNF suppressor element in the Osx promoter that was used to isolate nuclear proteins mediating TNF inhibition of OB differentiation. Nuclear extracts from TNF-treated pre-OBs were incubated with the TNF suppressor element for protein pull-down, and tryptic fragments were analyzed by mass spectrometry. Chromatin immunoprecipitation (ChIP) assay confirmed eight bound transcription factors. One protein, the paired related homeobox protein (Prx1), had been shown previously to have a critical role in limb bud formation and skeletal patterning. PCR revealed Prx1 expression in primary stromal cells (MSCs), C3H10T1/2 cells, and MC3T3 preosteoblasts. TNF stimulated a 14-fold increase in mRNA for Prx1, rapid cell accumulation in MC3T3 cells, and expression in periosteal and trabecular lining cells in vivo. Transient expression of Prx inhibited transcription of Osx and RUNX2. Expression of the Prx1b isoform or Prx2 decreased Osx and RUNX2 mRNA and OB differentiation in preosteoblasts. Silencing of Prx1 with siRNA abrogated TNF suppression of Osx mRNA and increased basal Osx expression. Electrophoretic mobility shift revealed Prx1b as the preferred isoform binding the Osx promoter. These results identify the homeobox protein Prx1 as an obligate mediator of TNF inhibition of Osx and differentiation of OB progenitors. Activation of Prx1 by TNF may contribute to reduced bone formation in inflammatory arthritis, menopause, and aging. © 2011 American Society for Bone and Mineral Research.

## Introduction

Osteoblasts are derived from pluripotent precursors of mesenchymal lineage. In adult bone, fracture healing and renewal recapitulate the developmental steps of bone formation, including recruitment and differentiation of new bone-forming osteoblasts (OBs).(1) OBs first must proliferate from a pluripotent precursor pool and then differentiate along a path toward a mature bone-forming phenotype rather than one of an adipocyte, myoblast, or fibroblast. Each step in the precursor commitment of an OB is orchestrated by expression of skeletal-specific transcription factors.(2–5) Critical transcription factors for OB differentiation include the runt-related factor (RUNX2, Cbfa1, AML3, and Pebp2αA2) and osterix (Osx, Sp7) because early knockout of these genes results in a cartilaginous skeleton.(6–10) The expression of RUNX2 and Osx is under regulation of soluble growth factors, cytokines, and systemic hormones. Several studies have shown a continued requirement for expression of skeletal transcription factors in adult bone.(11) We, and others, have hypothesized that inhibition of the expression of RUNX2 or Osx in inflammatory arthritis may impair bone formation and predispose to increased periarticular and systemic bone loss.(12–20)

Tumor necrosis factor α (TNF-α) has an important role as a mediator of skeletal damage in inflammatory arthritis.(14,21,22) TNF-α is now well established as a stimulus for the early recruitment of preosteoclasts from marrow progenitors and for expression of the receptor activator of NF-κB ligand (RANKL) and monocyte colony-stimulating factor (M-CSF) for subsequent genesis of bone-resorbing osteoclasts. In rheumatoid arthritis, TNF-α reaches high concentrations in the joint space during active disease, where it contributes to extensive tissue destruction.(23) A lower level of monocyte-derived TNF-α percolates in the bloodstream and contributes to systemic osteoporosis and increased fracture risk in inflammatory arthritis, menopause, and aging.(24–27) The mechanism of TNF-α as a mediator of bone resorption has been studied in detail, including activation of signaling pathways via NF-κB, MAPK, reactive oxygen species (ROS), and NFatC1 and the effect of these signals on steps in the oseoclast differentiation pathway.(28–30) Less is known regarding the inflammatory effects on OB function. A number of experimental models have been used to show an inhibitory role for TNF-α on OB differentiation and bone formation. These include TNF-α-expressing arthritic mice, alcohol suppression of bone formation, and estrogen deficiency.(31–42) We reported previously that TNF-α is a potent inhibitor of the skeletal transcription factors RUNX2 and Osx.(16,19) TNF-α inhibits *RUNX2* transcription by 50% and also stimulates a smurf1-mediated ubiquitination/degradation of *RUNX2*, leading to a 90% reduction in RUNX2 protein in vitro and in vivo.(43) TNF-α inhibits 90% of *Osx* transcription in a MAPK/ERK1/2-dependent process. Low concentrations of TNF-α also have been shown to inhibit the osteoblast-specific markers alkaline phosphatase, bone sialoprotein, and osteocalcin, as well as formation of mineralized matrix, at doses 100-fold lower than observed in the rheumatoid joint space, suggesting that OB precursors may be particularly sensitive to inhibition by TNF-α.(15,16,18) These actions of TNF-α impair the recruitment of new OBs to eroded areas and suppress healing of damaged bone in inflammatory disease.

We recently evaluated the structure of the Osx promoter to identify the mechanism of TNF-α inhibition.(19) Since regulation of Osx by TNF-α is entirely transcriptional, we took advantage of a defined TNF-α suppressor element in the Osx promoter to identify bound nuclear proteins that could be molecular mediators of inflammation and regulators of OB differentiation. Here we report the results of these studies and the finding that Prx1, a developmental regulator thought to be silenced after embryogenesis, is reactivated by TNF-α to mediate inhibition of osteoblastogenesis.

## Materials and Methods

### Reagents

MC3T3-E1 (clone 14) mouse preosteoblast cells were obtained from Dr Renny Franceschi (University of Michigan, Ann Arbor, MI, USA), and C3H10T1/2 cells were obtained from ATCC (Manassas, VA, USA). The sources of reagents were as follows: human TNF-α, PeproTech (Rocky Hill, NJ, USA); minimal essential medium (MEM) and α-MEM, Gibco/Invitrogen (Grand Island, NY, USA); heat-inactivated fetal bovine serum (FBS), HyClone (Logan, UT, USA); and trypsin/versene, phosphate-buffered saline (PBS, without Ca^2+^ and Mg^2+^), and the Amaxa Nucleofector device and reagents, Lonza (Walkersville, MD, USA). siRNA to Prx1, Prx2, and negative-control siRNA were from Ambion/Applied Biosystems (Austin, TX, USA), and siRNA transfection reagent was from Santa Cruz Biotechnology, Inc. (Santa Cruz, CA, USA). Superfect transfection reagent, RNeasy Micro Kit, and Taq PCR Core Kit were from Qiagen (Valencia, CA, USA). Oligonucleotides and primers were purchased from Eurofins MWG Operon (Huntsville, AL, USA). Real-time PCR was performed on the Bio-Rad MyiQ using the iScript cDNA Synthesis Kit and iQ SYBR Green Supermix (Hercules, CA, USA). Other reagents were obtained from Sigma-Aldrich (St Louis, MO, USA). The −1269/+91 Osx promoter-luciferase reporter (Osx–Luc) and deletion mutants were described previously.(19) The Dual Luciferase Assay System, TNF T7 Coupled Reticulocyte Lysate System, and pRL-TK control vector were purchased from Promega Corporation (Madison, WI, USA). Prx expression vectors were reported previously.(44) The Chromatin Immunoprecipitation (ChIP) Assay Kit was from Upstate Biotechnology/Millipore (Billerica, MA, USA). The p3xFLAG-CMV-14 vector (C-terminal 3xFLAG) was purchased from Sigma-Aldrich. Antibodies used for the ChIP assays were obtained from Santa Cruz Biotechnology. The RUNX2 promoter was obtained from Drs Jane Lian and Gary Stein (University of Massachusetts, Worcester, MA, USA) and described previously.(16,45) A rabbit antibody to Prx1 was raised to a 50-mer murine peptide in the laboratory of MJK and purified in the laboratory of MSN using sepharose chromatography,(46) The antibody bound Prx1a and Prx1b but did not bind Prx2, as revealed by Western blot analysis of recombinant proteins. Blocker bovine serum albumin (BSA) in PBS (10 × ) and the Fluorescein Isothiocyanate (FITC) Antibody Labeling Kit were from Pierce/Thermo Scientific, Inc. (Rockford, IL, USA). A second antibody to Prx1 was purchased from OriGene Technologies (Rockville, MD, USA), and a Prx2-specific antibody was purchased from Santa Cruz Biotechnology. Confocal microscopy was performed using a Fluoview 1000 microscope (Olympus Corporation, Center Valley, PA, USA).

### Nuclear pull-down

The oligo precipitation protocol was performed as described previously with minor modifications.(47) C3H10T1/2 cells were treated with TNF-α (10 ng/mL) or control medium for 18 hours. Cells were harvested in PBS and pelleted, and cytoplasmic and nuclear fractions were isolated.(47) Nuclear lysate (250 µg) from each sample was precleared with 75 µL of Dynal magnetic beads (Life Technologies, Carlsbad, CA, USA) and gel-shift binding buffer (Promega) for 30 minutes at room temperature. The Dynal MPC-S Magnetic Particle Concentrator was used to remove the beads. To bind the oligo to the beads, 100 µL of Dynal beads and Osx oligo (biotin-5'-TTGGATCTGAGTGGGAACAAGAGTGAGCTG-3' and 5'-CAGCTCACTCTTGTTCCCACTCAGATCCAA-3') were incubated at room temperature with gentle agitation for 30 minutes in PBS, followed by washing. Lysates from TNF-α and control samples were added to the oligo-labeled beads with gel-shift binding buffer (Promega) and incubated at room temperature for 45 minutes with gentle agitation. Beads were washed three times with binding buffer with increasing KCl concentrations (100, 250, and 500 mM) using the magnetic concentrator. The bound proteins were eluted from beads by adding 50 µL of 10 µM dithreothreitol (DTT) in 50 mM NH_4_ HCO_3_ solution and incubating at 100°C for 10 minutes. This elution was performed twice, and the supernatants were combined and lyophilized. The samples were reconstituted in 25 µL of 20% acetonitrile in 50 mM NH_4_ HCO^3−^ containing 0.4 µg of trypsin and incubated overnight at 37°C. Digests were lyophilized and desalted using PepClean C-18 spin columns (Pierce) and resuspended in 0.1% trifluoroacetic acid (TFA) prior to mass spectrographic (MS) analysis.

### Liquid chromatography mass spectrometry

Tandem mass spectrometry and bioinformatic analyses–liquid chromatography (LC) were performed using a Dionex UltiMate 3000 LC system (Dionex Corporation, Sunnyvale, CA, USA) coupled online with a linear-ion-trap (LIT) mass spectrometer (MS) (LTQ-XL, ThermoFisher Scientific, Inc., Waltham, MA, USA). Separations of each digest were performed on a 75-µm-inner-diameter × 360-µm-outer-diameter × 10-cm-long fused silica capillary column (Polymicro Technologies, Phoenix, AZ, USA), with 5-µm, 300-Å pore-size Jupiter C-18 stationary phase (Phenomenex, Torrance, CA, USA) with an integrated electrospray ionization (ESI) emitter tip. After injecting 5 µL of sample, the column was washed with 98% mobile phase A (0.1% formic acid in water) for 20 minutes, and peptides were eluted by development of a linear gradient of 2% mobile phase B (0.1% formic acid in acetonitrile) to 42% mobile phase B in 140 minutes and then to 98% mobile phase B in an additional 10 minutes, all at a constant flow rate of 250 nL/minute. The column was washed for 20 minutes with 98% mobile phase B and equilibrated with 98% mobile phase A prior to subsequent sample loading.

The MS was operated in a data-dependent MS/MS mode in which each full MS scan (precursor ion-selection scan range of *m/z* 350 to 1800) was followed by seven MS/MS scans, where the seven most abundant peptide molecular ions were selected for tandem MS using a relative collision-induced dissociation (CID) energy of 35%. Dynamic exclusion was used to minimize redundant selection of peptides for CID.

Tandem mass spectra were searched against the UniProt-derived *Mus musculus* proteome database (www.expasy.org) using SEQUEST (ThermoFisher Scientific, Inc., Waltham, MA, USA). Peptides were considered legitimately identified if they achieved a specific charge state and proteolytic cleavage–dependent cross-correlation (Xcorr) scores of 1.9 for [M + H]^+1^, 2.2 for [M + 2H]^+2^, and 3.1 for [M + 3H]^+3^ and a minimum delta correlation score (ΔCn) of 0.08. These stringent filters resulted in a false discovery rate of less than 1%, as determined by searching a reversed human proteome database, as described previously.(48)

### Cell culture

MC3T3-E1 clone 14 (MC3T3) or C3H10T1/2 cells were plated at 5 × 10^4^/mL per well in 12-well plates (Costar, Lowell, MA, USA) in MEM and 10% FBS, as described previously.(16) TNF-α was added on day 1 in the doses indicated for each experiment. Primary marrow stromal cultures (MSCs) were prepared as described using young-adult C57BL/6 male mice.(18)

### RNA harvest and quantitative RT-PCR

RNA was prepared using the RNeasy Micro Kit on the days indicated for each experiment. Osx and RUNX2 promoter-luciferase reporters were described previously.(16,19) Quantitation of mRNA in total cell RNA was done in duplicate or triplicate using the primers shown in Table 1.

**Table 1:** PCR Primers

Gene	Forward primer (5′→3′)	Reverse primer (5′→3′)
28S rRNA	TTGAAAATCCGGGGGAGAG	ACATTGTTCCAACATGCCAG
*Osx*	CCTCTCGACCCGACTGCAGATC	AGCTGCAAGCTCTCTGTAACCATGAC
*AP*	TTGTGCGAGAGAAAGAGAGAGA	GTTTCAGGGCATTTTTCAAGGT
*RUNX2*	GAATGGCAGCACGCTATTAAATCC	GCCGCTAGAATTCAAAACAGTTGG
*BSP*	AACGCCACACTTTCCACACTCT	CGTCGCTTTCCTTCACTTTTG
*OC*	CTGACAAAGCCTTCATGTCCAA	GCGCCGGAGTCTGTTCACTA
*Prx1*	CCCGGATGCTTTTGTTCGAGA	CATGTGGCAGAATAAGTAGCCAT
*Prx1a*	CATCGTACCTCGTCCTGCTC	AGTCTCAGGTTGGCAATGCT
*Prx1b*	CATCGTACCTCGTCCTGCTC	GCCCCTCGTGTAAACAACAT

### Transfection and reporter assays

For reporter assays, cells were transfected with a mixture of Superfect transfection reagent, medium, promoter reporter, and pRL-TK control vector. Cells were harvested 48 hours after transfection and assayed using the Dual Luciferase Assay System. Firefly luciferase values were normalized to *Renilla* luciferase data to correct for transfection efficiency. For transfection of expression vectors, the same technique was used with exclusion of *Renilla* luciferase vector. Addition of siRNA or randomized control was done 48 hours prior to treatment with TNF-α using siRNA transfection reagent. The medium was changed every 48 to 72 hours.

### In vitro translation

In vitro transcription/translation was done using the TNT T7 Coupled Reticulocyte Lysate System according to the manufacturer's instructions.

### Electrophoretic mobility shift assay

Electrophoretic mobility shifts (EMSAs) were done as described previously.(16,49) Probe sequences used for the TNF-α-responsive site and homeodomain-binding site of the murine Osx promoter are shown in the figures.

### Chromatin immunoprecipitation assay (ChIP)

ChIP assay was performed using the ChIP Assay Kit according to the manufacturer's instructions. Prx1 was cloned into the p3xFLAG-CMV-14 vector to create FLAG-Prx1 for use in the pull-down reactions. Antibodies used for ChIP assays were anti-HCC1, HSP70, Radixin, NF-κB p65, VDR, Moesin, TIF1α, FLAG, and normal mouse IgG. C3H10T1/2 cells were transfected with the Prx1-FLAG expression vector, treated with TNF-α (10 ng/mL), or control medium, and ChIP assay was done 24 hours later using 5 × 10^6^ C3H10T1/2 cells for each pull-down reaction. Detection of the antibody pull-down DNA complex of interest was done by PCR using 10 µL of DNA per sample and the Taq PCR Core Kit at 40 cycles of 95°C, 30 seconds; 60°C, 30 seconds; and 72°C, 1 minute. Primers spanned the TNF-α response region of the *Osx* gene and upstream or downstream control sequences. Primer sequences were TNF-α response region (forward) 5′-GACTC AGAAG GAATG ATAGA GGCT-3′ and (reverse) 5′-AGTTA CAATC TAGAG GCAGC CT-3′, upstream primers (forward) 5′-GTGTA TGTCA GTGTG TTCCA GTCTT-3′ and (reverse) 5′-GCTGG GAGGG ATCTG CTCCT CTCTC-3′, and downstream primers (forward) 5′-GGGTT AAGGG GAGCA AAGTC AGAT-3′ and (reverse) 5′-CTGGG GAAAG GAGGC ACAAA GAAG-3′. PCR products were resolved by agarose-gel electrophoresis and visualized with ethidium bromide.

### Fluorescent immunohistochemistry

MC3T3 cells were treated with control medium or TNF-α (10 ng/mL), as indicated in the figures. Cells were fixed in 95% ethanol for 10 minutes, followed by rinsing three times in PBS for 5 minutes each. Cells were blocked using 3% bovine serum albumin (BSA) in PBS (prepared from Blocker BSA in PBS) for 1 hour at room temperature. Prx1 antibody labeled with fluorescein isothiocyanate (FITC) was prepared according to kit instructions. Endogenous Prx1 was detected by incubating fixed cells with FITC–anti–Prx1 (1:50 in PBS) at 4°C overnight, followed by rinsing two times in PBS for 5 minutes each. Nuclei were counterstained with 4,6-diamidino-2-phenylindole (DAPI), and cells were examined using a confocal microscope with a ×40 objective under oil immersion. Wavelengths used were 488 (FITC) and 350 nm (DAPI).

### TgTNF mice and histology

Use of mice was approved by the institutional animal use committee of the VA Medical Center. Female TgTNF mice [B6.Cg(SJL)-Tg(TNF) N21 + ?] were purchased from Taconic (Hudson, NY, USA). Tibias were harvested from 12-week-old mice expressing 3 to 5 pg/mL of human TNF-α or from wild-type littermates. Bones were cleaned, fixed in paraformaldehyde, and embedded in paraffin, and 5-µm cuts of the proximal tibia were made to include a portion of the cartilage, trabecular bone, and periosteal bone. Sections were processed for immunohistology using the antibody raised in our laboratory and described earlier.

### Statistical analysis

ANOVA was used to determine a statistical difference between multiple groups. Multiple comparisons between individual groups were done by the method of Tukey. Comparison between any one group and a common control was done by the method of Dunnett using Prism software (Irvine, CA, USA).

## Results

### Identification of proteins binding the TNF-suppressor element of the Osx promoter

The TNF-responsive sequence from the Osx promoter, TTGGATCTGAGTGGGAACAAGAGTGAGCTG, was bound to sepharose beads and used as bait to identify the interacting nuclear protein complex in MC3T3 cells. MS identified fragments from 655 individual proteins. Since the probability of a true positive TNF-regulated protein increases with the number of identified fragments of that protein, further analysis was restricted to 62 proteins for which 2 or more fragments were detected and TNF modified detection of the protein. Table 2 shows these proteins arranged by known function. As expected, most were nuclear matrix, histone, ribonuclear, or ribosomal-binding proteins associated with nuclear structure and its organization around the transcriptional apparatus. Eight proteins were studied further because they were transcription-modifying nuclear proteins, regulators of cell differentiation, or known TNF-regulated proteins. Analysis focused on these as potential direct regulators of the Osx promoter. Three of the proteins were TNF-displaced. These included cysteine-rich protein-2 (CRP-2, DLP-2), known to regulate differentiation of smooth muscle from mesenchyme; HCC-1, a DNA-binding factor that inhibits cell growth; and moesin, a member of the “merlin” regulatory complex. Five proteins had increased binding after TNF-α treatment. These included tripartite motif protein 28 (TIF1β), a known transcriptional coactivator of nuclear receptors; heat shock cognate 71-kD protein, a chaperone previously identified in an array of TNF-induced proteins; radixin (ESP-10), another component of the “merlin” complex of ezrin-moesin-radixin described to block NF-κB-induced transcription; Ewing sarcoma homologue; and paired mesoderm homeobox protein 1 (Prx1, Prrx-1, MHox). Prx1 is a factor required for early limb bud formation, skeletal patterning, and early pulmonary and cardiovascular differentiation.

**Table 2:** Nuclear Factors Identified by Mass Spectroscopy at the Osx TNF-α Element With Known Differentiation Regulatory, Transcriptional Regulatory, or TNF-α-Responsive Functions

Factor	Known functions	Accession number
TNF-induced		
TIF1β Tripartite motif protein 28	Transcriptional coactivator of nuclear receptors	Q62318
HSC71 Heat shock cognate 71-kDa protein	TNF-induced chaperone protein	P63017
Radixin (ESP-10)	Component of “merlin” complex; regulation of NF-κB-induced transcription, actin-modifying protein	P26043
Ewing sarcoma homologue	Associates with TFIID	Q5SUS9
PRX1 Paired mesoderm homeobox protein 1	Early limb bud, bone, and vascular differentiation factor	P63013
TNF-displaced		
CRP-2 Cysteine-rich protein 2 (DLP-2)	Mesenchyme smooth muscle differentiation factor; LIM protein expressed e9.5-e16.5 in smooth muscle	P97315
HCC-1	DNA-binding factor, inhibits cell growth	Q9D1J3
Moesin Membrane-organizing extension spike protein	Component of “merlin” complex; regulation of NF-κB-induced transcription, actin-modifying protein	P26041

**Fig. 1:**
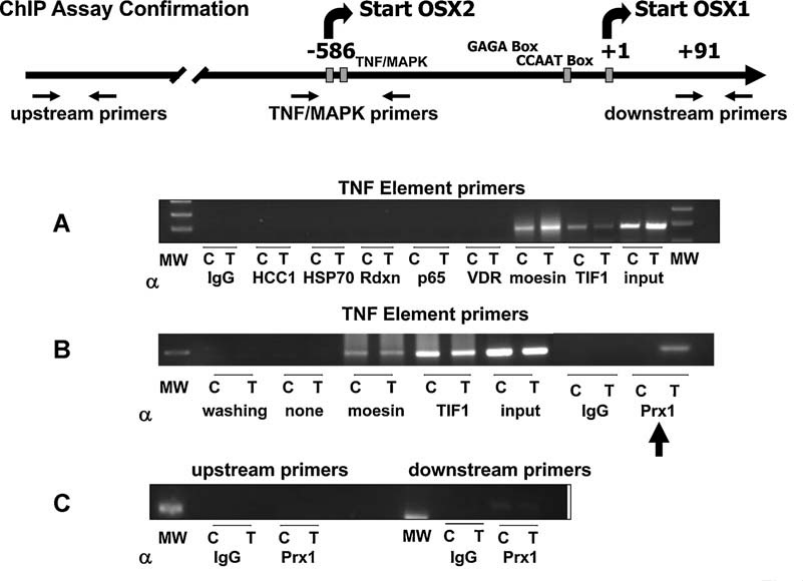
Protein binding to the TNF-α repressor element of the Osx promoter is confirmed by ChIP assays. C3H10T1/2 cells treated with TNF-α (10 ng/mL) or control medium for 24 hours were used for ChIP assays. At the top is a map of the Osx promoter, including the TNF-α response element region. Primer sequences are listed in Table 1. (*A*) ChIP assays used antibodies against normal mouse IgG (as control), HCC1, HSP70, radixin, NF-κB p65, VDR, moesin, or TIF1. PCR reactions used the primer pair flanking the TNF-α response element region indicated on the map. Molecular-weight markers are on each end. (*B*) ChIP assays used antibodies against normal mouse IgG (as control), moesin, TIF1, or Prx1. Other controls were done to test the wash step and the effect of no antibody, as well as to verify the presence of the relevant sequence in the starting DNA sample (input). The primers used were the same as in panel *A*. (*C*) PCR reactions used the same DNA as in panels *A* and *B* and primers that were upstream or downstream of the TNF-α response element.

ChIP assays were done to confirm binding of the selected proteins to the TNF-α repressor element of the Osx promoter. C3H10T1/2 cells were used for ChIP assays after treatment with TNF-α (10 ng/mL) or control medium. These experiments confirmed binding of moesin, TIF1β, and Prx1 to the region containing the TNF-α element. Figure 1 shows a map of the Osx promoter and illustrates the location of TNF-α element–flanking primers used for ChIP assay. The experiment shown in Fig. 1*A* did not support binding of HCC1 or radixin with the available antibodies. As expected, the negative controls IgG, p65, and VDR were not bound. Figure 1*B* shows that TNF-α stimulated the binding of Prx1 (*arrow*) and confirmed the constitutive binding of moesin and TIF1β. Figure 1*C* shows that upstream and downstream primers, distal to the TNF/MAPK element, revealed only a faint signal or none, supporting the specificity of Prx1 bound to the TNF-α site.

### TNF-α regulates Prx expression

**Fig. 2:**
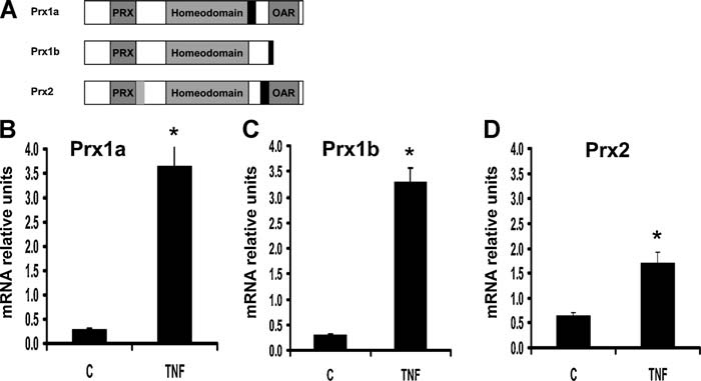
Prx expression is regulated by TNF-α. (*A*) Gene structures of the Prx1 isoforms and Prx2. (*B–D*) RNA was prepared from C3H10T1/2 cells treated with control medium or TNF-α (10 ng/mL) for 24 hours. Quantitative RT-PCR using primer pairs specific for Prx1a (*B*), Prx1b (*C*), or Prx2 (*D*) (Table 1) was performed.

Since TNF-α stimulated binding of Prx1, a factor previously described as a regulator of skeletogenesis, we focused on this factor as a potential mediator of TNF-α suppression of *Osx* transcription. Figure 2 shows the effect of TNF-α treatment on the expression of Prx1a and Prx1b isoforms, as well as the closely related *Prx2* gene. The comparative structures of Prx1 isoforms and Prx2 are shown in Fig. 2*A*, as adapted from Norris and colleagues.(44) These proteins share a common Prx region and homeodomain sequence. Prx1a and Prx2 also share a similar transcriptional activation domain (OAR). TNF-α robustly stimulated the expression of Prx1a and Prx1b 14-fold (Fig. 2*B, C*). TNF-α also modestly stimulated Prx2 2.8-fold (Fig. 2*D*).

**Fig. 3:**
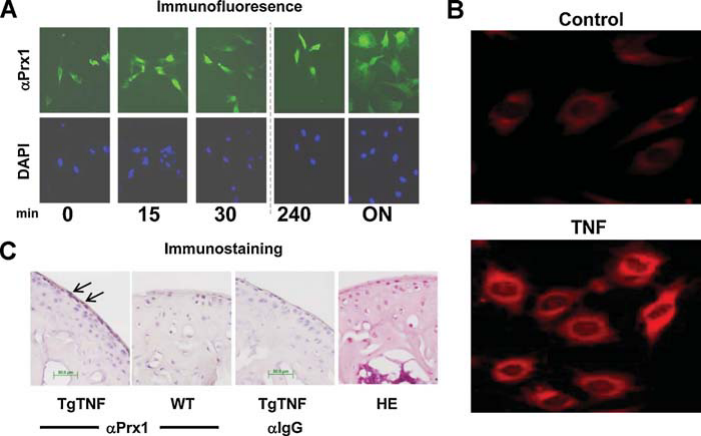
TNF-α stimulates expression of Prx1 in vitro and in vivo. (*A*) MC3T3 cells treated with control medium or TNF-α (10 ng/mL) for the indicated times and labeled with fluorescein-conjugated anti-Prx1 antibody (FITC-Prx-1). Increased signal intensity is seen within 15 minutes and is sustained. Nuclei were counterstained with DAPI, and cells were examined by confocal microscopy with a ×40 oil-immersion objective. (*B*) As in panel *A*, showing a closer view of increased cytoplasmic and punctuate nuclear accumulation after TNF-α treatment from another experiment. (*C*) Immunostaining shows Prx detection in tibial periosteal lining cells from TgTNF arthritic mice but little or no signal in wild-type littermates. Shown are TgTNF, wild-type, control antibody, and hematoxylin and eosine (H&E)–stained sections from the proximal tibia. Bones were obtained from 8-week-old mice.

Treatment of undifferentiated MC3T3 cells with TNF-α stimulated the accumulation of Prx1 protein in the cytoplasm and nucleus within 15 minutes, as shown by fluorescent immunohistology (Fig. 3*A*). This nuclear localization was sustained through a subsequent 18 hours of culture (ON = overnight). Figure 3*B* is a closer view from another experiment showing cytoplasmic and punctuate nuclear accumulation after TNF-α treatment. To determine if Prx1 is expressed in vivo, we used transgenic TNF mice (TgTNF) that develop inflammatory arthritis and systemic bone loss in response to global expression of human TNF. Immunostaining of bones obtained from 12-week-old mice revealed Prx1 staining in periosteal lining cells that was only weakly detected in bones of wild-type littermates (Fig. 3*C*). Additional sections revealed staining in trabecular lining cells and in macrophages and megakaryocytes in bone marrow (not shown).

### Prx1 mediates TNF-α inhibition of *Osx* transcription

**Fig. 4:**
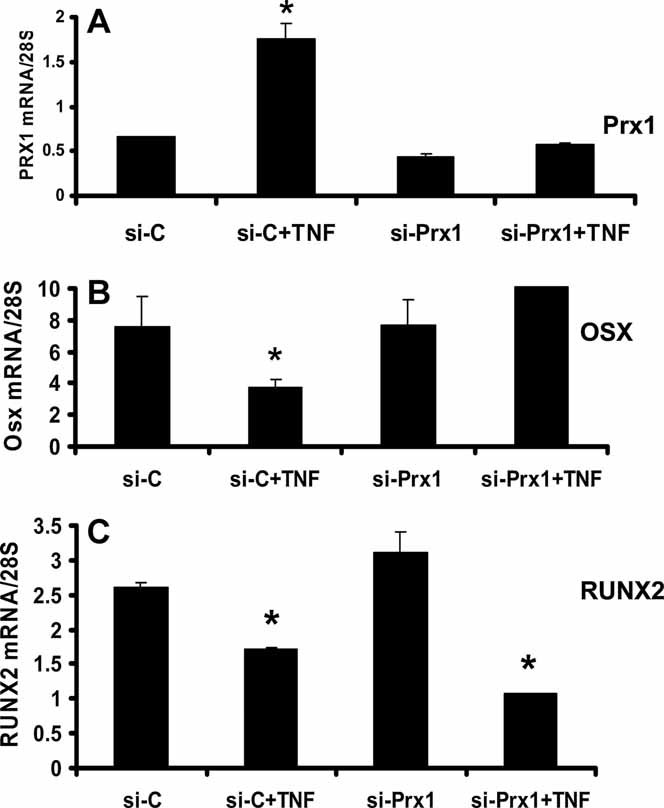
TNF-α inhibition of *Osx* mRNA is mediated by Prx1. C3H10T1/2 cells were transfected with control or *Prx1* siRNA, followed by treatment with control medium or TNF-α (10 ng/mL) for 24 hours. RNA was prepared and analyzed by quantitative RT-PCR using Prx1-, Osx-, or Runx2-specific primer pairs, as shown in Table 1. (*A*) Prx1. (*B*) Osx. (*C*) Runx2.

We tested the role of Prx1 as a mediator of TNF-α inhibition of *Osx* by silencing *Prx1* with a short interfering RNA (si-Prx1) that inhibited expression of all Prx1 isoforms. Figure 4*A* shows that TNF-α stimulation of Prx1 was blocked by the si-Prx1. si-Prx1 had no effect on Prx2 expression (not shown). Figure 4*B* shows that the si-Prx1 completely abrogated TNF-α inhibition of *Osx* mRNA. Blockade of TNF-α inhibitory action by si-Prx1 was not observed for *RUNX2* mRNA (Fig. 4*C*).

### Prx1b and Prx2 bind the TNF-α element and an upstream homeobox site

**Fig. 5:**
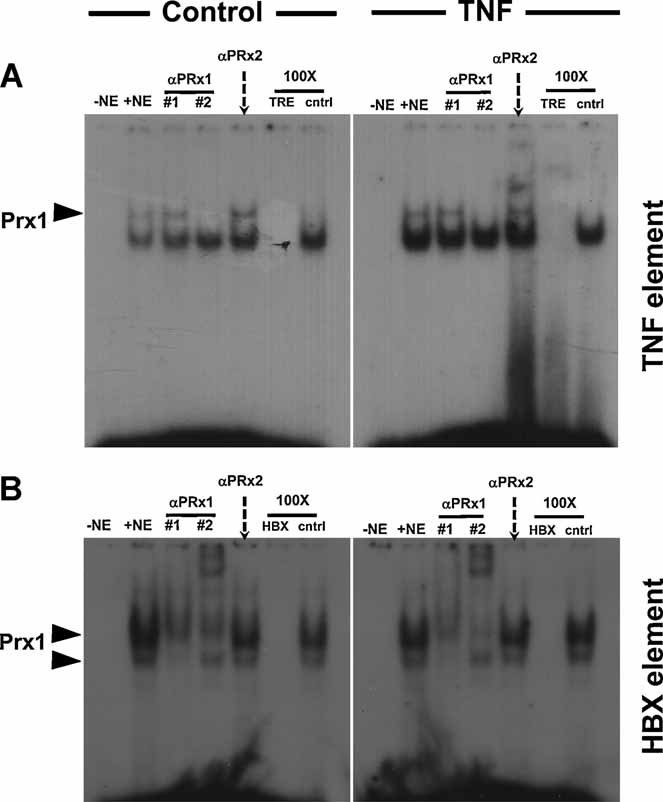
Prx1 binds the Osx promoter. Nuclear extract was prepared from C3H10T1/2 cells treated with TNF-α (10 ng/mL) for 18 hours. EMSA was carried out using ^32^P-labeled dsDNA TNF-α response element or homeobox-binding site oligos, along with the indicated combinations of nuclear extract, antibodies, and cold probes. (*A*) EMSA showing labeled TNF-α response element–binding nuclear protein from control or TNF-α-treated C3H10T1/2 cells. The arrow on the left of the gel identifies the Prx1 band. TNF-α increased binding to the probe (control, +NE versus TNF, +NE). The binding is supershifted by a Prx1 antibody (αPrx#2) but not by a Prx2 antibody (αPrx2) or a commercial Prx1 antibody (αPrx1#1) and competed by 100X unlabeled probe (TRE) but not by 100X unlabeled control probe. (*B*) EMSA using a probe from a homeobox homologous site upstream of the TNF-α site that includes a contiguous RUNX2-binding site. The arrows on the left of the gel identify Prx1 bands. The binding is reduced or supershifted by two different Prx1 antibodies (αPrx1, #1 or #2) and competed by 100X cold probe. TNF-α treatment did not change the binding to this site.

Analysis of the Osx promoter sequence revealed a homologous homeobox-binding site just upstream of the 18-bp TNF-α response element, contiguous with a previously described RUNX2-binding site.(50) Therefore, we evaluated whether TNF stimulated binding of Prx1 to the Osx promoter at both the TNF-α response element and the upstream homeobox-RUNX2 site. Nuclear extract from C3H10T1/2 cells was isolated from control and TNF-α-treated cells and incubated with ^32^P-labeled TNF-α response element or homeobox probes. Figure 5*A* shows that two bands were observed at the TNF-α element (*A*, control, +NE) that increased in intensity after 18 hours of TNF-α treatment (*A*, TNF, +NE). This complex contained Prx1 because the top band was supershifted by our Prx1 antibody (αPRX1#2) but not by a commercial antibody (αPRX#1) or a Prx2 antibody (αPRX2). Specificity of binding also was demonstrated by competition with 100X cold TNF element probe (TRE) but not by a control DNA sequence. Figure 5*B* shows that the homeobox probe also bound a complex that was supershifted by both Prx1 antibodies but not by the Prx2 antibody. This binding was specific because it was competed by 100X unlabeled homeobox probe (HBX). In contrast to the effect of TNF-α on binding of Prx1 to the TNF-α element, binding to the homeobox site was constitutive.

**Fig. 6:**
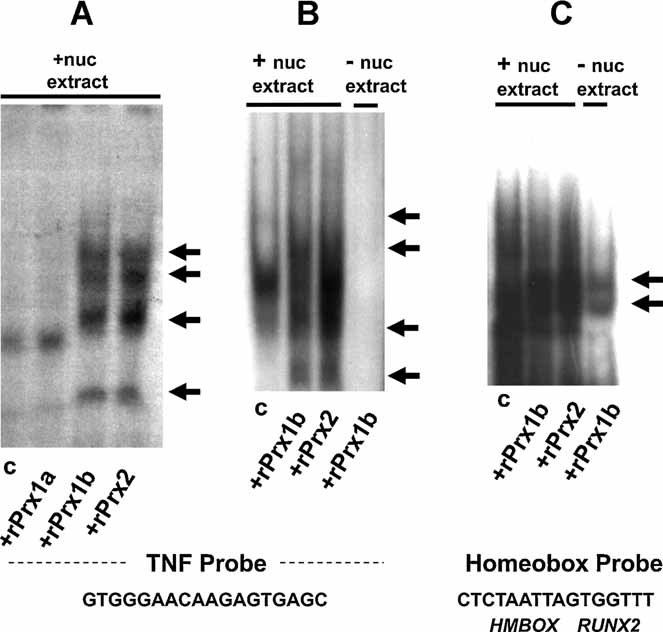
Delineation of Prx isoforms binding the Osx promoter TNF-α response element. Recombinant Prx1a, Prx1b, and Prx2 proteins were synthesized by in vitro transcription/translation. Nuclear extract was prepared from C3H10T1/2 cells treated with TNF-α (10 ng/mL) for 24 hours. EMSA was carried out using ^32^P-labeled dsDNA TNF-α response element or homeobox-binding site oligos, along with the indicated combinations of recombinant proteins and nuclear extract. Controls were prepared without recombinant protein. (*A*) Recombinant Prx1b and Prx2, but not Prx1a, bind the TNF-α response element in the presence of nuclear extract. (*B*) Recombinant Prx1b fails to bind the TNF-α response element in the absence of nuclear extract. (*C*) Homeobox-binding site (upstream of the TNF-α element). Recombinant Prx1b and Prx2 bind the upstream homeobox element with or without nuclear extract. Arrows indicate the locations of protein-DNA complexes.

Binding to the promoter was evaluated further with recombinant Prx1a and Prx1b. Proteins were synthesized by in vitro transcription/translation using isoform-specific cDNA templates driven by the bacterial T7 promoter. These protein products or a control reaction supernatant was tested for binding to the TNF-α element of the Osx promoter. Figure 6*A* shows an EMSA done with the ^32^P-labeled TNF-α element, the recombinant proteins, and nuclear extract from TNF-α-treated C3H10T1/2 pluripotent mesenchymal cells. Prx1b and Prx2, but not Prx1a or the control reaction, readily bound the probe under these conditions. Binding of recombinant Prx1b required the inclusion of nuclear extract in the EMSA reaction, as shown in Fig. 6*B*. Figure 6*C* shows that Prx1b was able to bind the homeobox site in the presence or absence of nuclear extract.

### Prx1b and Prx2 inhibit Osx and RUNX2 expression and osteoblast differentiation

**Fig. 7:**
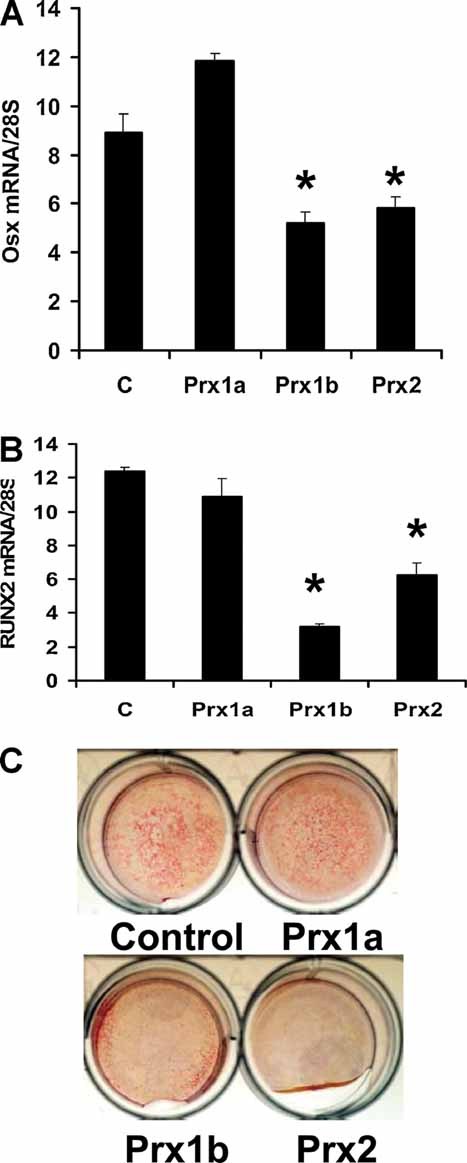
Osteoblast differentiation and Osx and RUNX2 expression are inhibited by Prx1b and Prx2 in preosteoblasts. MC3T3 cells were transiently transfected with Prx1a, Prx1b, or Prx2 expression vectors using the Amaxa nucleofection system. RNA was prepared 48 hours after transfection. (*A*) Quantitative RT-PCR using primers specific for *Osx*. (*B*) Quantitative RT-PCR using primers specific for *RUNX2*. (*C*) MC3T3 cells transiently transfected with Prx1a, Prx1b, or Prx2 expression vectors using the Amaxa nucleofection system. Transfected cells were cultured in mineralization medium for 14 days and then stained with alizarin red.

**Fig. 8:**
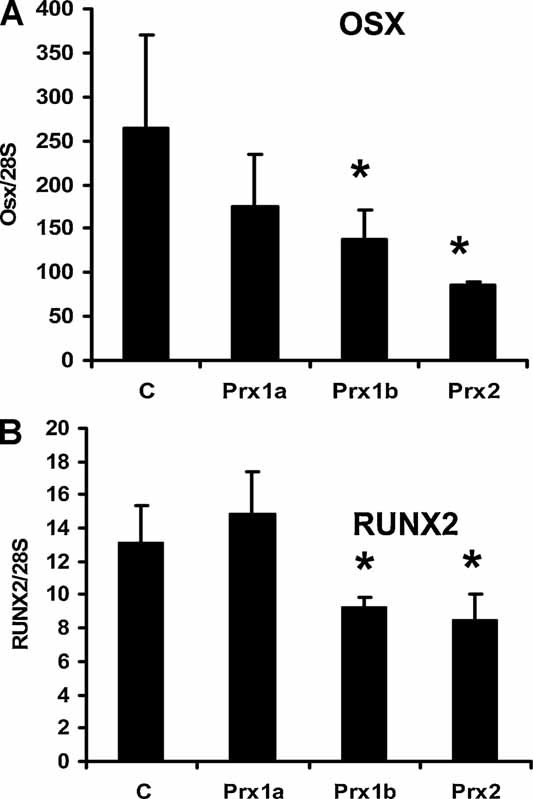
Prx1b and Prx2 inhibit osteoblast differentiation and Osx and RUNX2 expression in primary MSC cultures. Mouse primary MSC suspensions were prepared at 5 × 10^6^ cells/mL in α-MEM and 10% FBS and transiently transfected with Prx1a, Prx1b, or Prx2 expression vectors using the Amaxa nucleofection system. Transfected cells were plated in 12-well plates (1 mL/well) and supplemented with 50 &g/mL of l-ascorbate and 5 mM β-GP. RNA was prepared after 48 hours, and quantitative RT-PCR was performed. (*A*) *Osx*. (*B*) *RUNX2*.

We tested whether Prx expression would inhibit the expression of the genes required for OB differentiation or the expression of phenotypic markers of the mature OB. MC3T3 cells or primary MSCs were transiently transfected with Prx1a, Prx1b, or Prx2 expression vectors. Figure 7*A* shows that overexpression of Prx1b and Prx2, but not Prx1a, inhibited *Osx* mRNA levels by 50%, similar to the action of TNF-α.(19) Interestingly, although silencing of Prx1 did not affect basal *RUNX2* mRNA, overexpression of Prx1b and Prx2 inhibited *RUNX2* mRNA (Fig. 7*B*). Expression of Prx proteins also reduced transcription of both Osx- and RUNX2-luciferase reporters, with Prx2 showing the highest potency (not shown). Transient expression of Prx1b and Prx2 also inhibited terminal differentiation of MC3T3 cells, as seen by the reduction in mineralized alizarin red–stained nodules in Fig. 7*C*. Figure 8*A*, *B* shows a similar experiment in primary MSCs. Again, Prx1b and Prx2, but not Prx1a, inhibited *Osx* and *RUNX2* (Fig. 8*A, B*).

## Discussion

Our experiments were designed to reveal early steps in the molecular action of TNF-α that inhibit OB differentiation. A critical requirement in OB differentiation is the expression of Osx because lack of this protein during development results in a cartilaginous skeleton.(10) Since TNF-α inhibits Osx expression by a predominantly transcriptional mechanism, we focused on identification of TNF-α-induced proteins bound to the core TNF-α inhibitory element on the Osx promoter. Our pull-down studies identified a number of proteins that were bound to this DNA element in a TNF-α-dependent manner. One of these, Prx1, was regulated by TNF-α and confirmed in ChIP assay. We have not excluded a role for other proteins, including moesin and TIF1β, which could cooperate with Prx1; however, Prx1 was induced by TNF-α treatment, and functional studies revealed a specific role for this protein.

We found that TNF-α potently upregulates *Prx1* mRNA and protein with a lesser stimulation of the closely related *Prx2* in cultured primitive mesenchymal cells. In addition, TNF-α stimulated rapid nuclear localization of Prx protein and positive immunostaining in bone lining cells that are a source of preosteoblasts. Functional studies support a role for Prx as a mediator of TNF-α. First, silencing of *Prx1* using a Prx1-specific si-RNA abrogated TNF-α inhibition of Osx expression. Second, overexpression of Prx1b and Prx2, but not Prx1a, inhibited *Osx* and *RUNX2* mRNA. Finally, Prx1b and Prx2 transient expression inhibited the osteoblastic differentiation of preosteoblasts. These results suggest that Prx1b and Prx2 are TNF-α-induced inhibitors of Osx and OB differentiation.

*Prx* genes have long been known to have a role in embryonic limb bud formation and skeletal development.(51–54) Prx1 is expressed in developing mouse mesenchyme until day e11.5, a time spanning the onset of skeletal development, after which levels are reduced. Prx1 also indirectly modulates sonic hedgehog expression, a factor important for the balance of proliferation versus differentiation of precursor cells.(55) The finding that Prx1 mediates TNF-α inhibition of OB differentiation is consistent with previous reports on the effect of global deletion of *Prx1* or *Prx2*. An abnormal skeletal phenotype is observed in *Prx1*^−*/*−^, but not *Prx2*^−*/*−^, mice, characterized by defects in mandibular development, short, thickened limbs, and normal growth plates.(53) Although *Prx2* knockouts are indistinguishable from wild-type mice, *Prx1*^−*/*−^/*Prx2*^−*/*−^ double knockouts have a more severe phenotype, including defects of the craniofacial, limb, and vertebral skeletons. These findings indicate a weak functional redundancy of Prx1 and Prx2. Although *Prx* genes were first identified as limb bud inducers, sustained expression could serve to limit premature bone maturation to ensure correct skeletal patterning. Thus, silencing of *Prx* expression may be required to allow skeletal maturation to proceed coordinately with limb development. This hypothesis is consistent with in vitro studies of mesenchymal differentiation in micromass cultures showing Prx1b inhibition of chondrogenic nodule formation.(56)

We found that TNF-α stimulated the expression of Prx1. TNF-α induces Prx1 more robustly than Prx2, suggesting that it is the more important mediator of inflammation. Information regarding the role of Prx1 in bone is limited because global knockout is lethal owing to abnormal cardiac and pulmonary vascular development. Although the specific role of Prx1 in OB differentiation has yet to be unveiled, our results indicate that TNF-α, and perhaps other inflammatory stimuli, could reactivate Prx expression in inflammatory arthritis, aging, and menopause. This reactivation of Prx1 then would decrease Osx and RUNX2, reduce OB differentiation, and impair healing of resorbed bone.

Our studies also suggest that Prx1b is the isoform that mediates TNF-α inhibition of Osx and OB differentiation. We found that recombinant Prx1b, but not Prx1a, binds the TNF-α element of the Osx promoter in EMSA. Similarly, expression of Prx1b, but not Prx1a, inhibits OB differentiation in vitro. Prx isoforms have been shown previously to differentially regulate transcription. Transcriptional potency has been attributed to a conserved Prx domain among homeobox-containing genes, whereas an OAR (aristaless domain, conserved among paired-type homeodomain proteins), present in Prx1a, but not Prx1b, has been found to be an inhibitor of *tenacsin* gene transcription.(44) The OAR is unlikely to account for the difference between Prx1a and Prx1b in our studies because it is conserved in Prx2, which functions similarly to Prx1b as an OB inhibitor. EMSA studies showed that recombinant Prx1b bound the TNF-α element protein complex on the Osx promoter and that this binding greatly increased after TNF-α treatment. We predict that this binding could be stabilized by the upstream homeobox site, which also was found to bind Prx constitutively. ChIP assay is not sensitive enough to precisely localize the binding of Prx1 because the primers span too large a region to detect the PCR signal. Nevertheless, the TNF-α element is sufficient for TNF-α action because point mutations of this sequence abolish TNF-α inhibition of *Osx* transcription, and transfer of the TNF-α element to a heterologous promoter confers TNF-α responsiveness in the absence of the upstream homeodomain sequence.(19)

Our prior studies of the Osx promoter revealed a MAPK/MEK/ERK1/2-dependent mechanism for TNF-α action. Although additional studies will be required to determine if TNF-α activation of Prx1b involves MAPK, experiments were reported to show MAPK/MEK/ERK1/2 and also AKT dependence for Prx action in amphibian regenerating limbs.(57)

TNF-α has a major role in the pathophysiology of skeletal disorders, including inflammatory arthritis, menopausal bone loss, and aging. These disorders share a reduced capacity of OBs to balance bone formation with resorption. Insufficient OB differentiation and function are likely reasons for this defect. We and others established TNF-α as an inhibitor of early commitment of OB precursors.(13,14,16,17,56) In rheumatoid arthritis, OB function is impaired, and continued periarticular bone destruction is not balanced by adequate bone formation, an effect countered by anti-TNF-α therapy.(17,58,59) In another model of decreased bone formation owing to alcohol exposure, TNF-α is responsible for inhibiting the recruitment of OBs needed for skeletal renewal.(34–38) Ovariectomy is associated with a rise in the expression of inflammatory cytokines, including TNF-α, accelerated bone resorption, and a blunted increase in bone formation that is insufficient to prevent net bone loss. Finally, recent work suggests that impaired fracture healing in aging is due to reduced OB differentiation and function, a defect corrected by inhibition of TNF-α.(38) Thus in vitro and in vivo skeletal models show that TNF-α suppresses OB differentiation and bone formation, in addition to stimulation of osteoclastic resorption. In summary, we have identified Prx1 as a molecular mediator of TNF-α inhibition of OB differentiation through suppression of Osx and RUNX2 expression. Further studies will be needed to evaluate the role of Prx1 in vivo.
